# Supplementary material for: The Effects of Pavement Types on Soil Bacterial Communities across Different Depths
Source: Int J Environ Res Public Health. 2019 May 21;16(10):1805. doi: 10.3390/ijerph16101805 (PMC6571668; doi:10.3390/ijerph16101805)
Supplement: Supplementary file 1 [file ijerph-16-01805-s001.pdf]

International Journal of
Environmental Research and Public Health

MDPI

Article

The Effects of Pavement Types on Soil Bacterial Communities across Different Depths

Weiwei Yu, Yinhong Hu, Bowen Cui, Yuanyuan Chen and Xiaoke Wang

Table S1. The two-way analysis variance of soil physical and chemical properties across land pavement. Data are expressed as mean ± SE, n = 3. SMC: soil moisture content; TC: total carbon; SOC: soil organic carbon; TN: total nitrogen; DOC: dissolved organic carbon; AP: available phosphorus; AK: available potassium. PP: pervious pavement; IPP: impervious pavement; NP: non-pavement.

Depth	Pavement	pH	g/kg			mg/kg			
			TC	TN	SOC	NH_4^+-N	NO_3^--N	AP	AK
0-20	IPP	6.86±0.03	14.95±1.15	0.87±0.06	9.34±0.83	12.69±0.28	20.21±9.29	26.6±5.36	82.36±12.04
	PP	6.75±0.14	14.26±0.38	0.83±0.06	6.52±1.10	12.37±0.28	5.01±0.28	50.42±13.05	128.32±17.40
	NP	7.03±0.05	16.66±0.74	0.99±0.08	8.27±0.54	12.7±0.14	8.93±0.87	61.67±2.54	84.53±7.66
20-40	IPP	6.87±0.03	12.39±1.02	0.65±0.12	6.26±1.70	12.45±0.31	26.05±12.71	11.16±5.57	61.78±12.31
	PP	6.78±0.08	13.64±1.29	0.6±0.11	5.10±1.30	12.35±0.41	7.69±5.81	26.47±17.27	79.6±24.09
	NP	7.00±0.07	13.57±1.31	0.75±0.12	6.63±1.11	12.72±0.13	11.66±2.45	40.29±9.72	65.13±4.15
40-60	IPP	6.87±0.06	12.00±1.35	0.64±0.14	6.53±1.29	12.48±0.26	50.99±26.87	7.58±8.15	71.43±20.10
	PP	6.82±0.22	13.57±2.06	0.54±0.05	4.42±1.00	12.3±0.20	5.31±2.62	3.40±2.80	64.91±23.56
	NP	7.13±0.04	11.54±1.07	0.53±0.05	4.05±0.08	12.39±0.19	8.18±2.61	4.54±1.70	72.36±8.10
60-80	IPP	6.81±0.06	11.66±2.43	0.72±0.13	7.85±0.87	12.23±0.37	57.13±26.64	8.90±10.17	92.14±28.23
	PP	6.79±0.24	12.09±3.15	0.51±0.04	4.05±0.88	11.98±0.16	6.13±1.82	2.26±0.61	66.77±24.44
	NP	7.16±0.01	12.58±1.69	0.56±0.04	4.87±0.59	12.41±0.41	8.07±2.54	2.67±1.47	87.83±20.66
80-100	IPP	6.89±0.05	9.88±2.12	0.66±0.07	6.75±0.84	11.78±0.26	37.41±4.63	2.53±1.64	100.31±10.69
	PP	6.84±0.11	11.39±2.2	0.58±0.03	5.24±0.49	11.81±0.14	18.05±2.22	1.94±0.17	84.98±9.78
	NP	7.14±0.02	12.1±0.49	0.61±0.02	6.28±1.04	13.06±1.77	7.10±1.00	3.01±1.96	102.59±1.64
Two-way ANOVA									

Pavement	**	ns	*	**	ns	**	**	ns
Depth	ns	**	**	**	ns	ns	**	**

* *P* <0.05; ** *P* <0.01.

Table S2. Pearson's correlation between soil characteristics and the relative abundance of abundant phyla.

	Actinobacteria	*Chloroflexi*	*Proteobacteria*	*Acidobacteria*	*Nitrospirae*	*Gemmatimonadetes*	*GAL15*	*Firmicutes*	*Planctomycetes*
pH	–	-0.362*	0.371*	-0.317*	–	–	–	–	-0.425**
TC	0.302*	–	0.310*	–	-0.649**	–	-0.595**	0.376*	-0.446**
TN	–	–	–	–	0.495**	–	-0.540**	–	–
SOC	–	–	–	–	–	–	-0.343*	–	–
NH_4^+-N	0.36*	-0.313*	–	–	–	–	–	–	–
NO_3^--N	–	0.406**	-0.370*	–	–	–	–	–	0.406**
AP	0.312*	–	0.336*	–	-0.633**	–	-0.651**	0.298*	–
AK	–	–	–	0.307*	–	–	–	–	–

* *P* <0.05; ** *P* <0.01.

© 2019 by the authors. Submitted for possible open access publication under the terms and conditions of the Creative Commons Attribution (CC BY) license (http://creativecommons.org/licenses/by/4.0/).
